# Thyroid Disorders in Saudi Patients With Acromegaly: A Tertiary Care Center Experience

**DOI:** 10.7759/cureus.53663

**Published:** 2024-02-05

**Authors:** Moayad A Alsuraikh, Eyad Almalki, Tuqa Bazuhair, Mussa Almalki

**Affiliations:** 1 Obesity, Endocrine, and Metabolism Center, King Fahad Medical City, Riyadh, SAU; 2 College of Medicine, Shaqra University, Shaqra, SAU; 3 College of Medicine, Alfaisal University, Riyadh, SAU

**Keywords:** thyroid autoimmunity, thyroid ultrasonography, igf-1, gh, thyroid nodules, cancer, goiter, thyroid dysfunction, acromegaly

## Abstract

Background

Acromegaly is a rare disease that is frequently associated with thyroid diseases. The exact prevalence of goiter and thyroid dysfunction remains uncertain.

Objectives

This study aims to provide a comprehensive description of the clinical, morphological, and biochemical features of thyroid disorder in Saudi patients with acromegaly and to establish its correlation with the activity and duration of the disease.

Methods

This retrospective study involved patients who were diagnosed with acromegaly during the period 2006-2023 in an outpatient endocrine clinic at a tertiary hospital.

Results

A total of 40 patients with acromegaly (27 males and 13 females) were identified and included in the analysis, with a mean age of 46.78 ± 13.76 years and an estimated duration of disease of 8.08 ± 6.43 years. Goiter was diagnosed in 28 patients (70.0%), including multinodular goiter (MNG) (70.0%), solitary thyroid nodules (14.2%), and thyroid cysts (14.2%). Primary hypothyroidism was present at 40.0%. Goiter was not correlated with estimated insulin-like growth factor 1 (IGF-1) levels or disease duration. In 40 patients with nodular goiter, fine needle biopsies were performed in six cases; five nodules were benign, and one nodule was a follicular lesion of unknown significance (Bethesda III).

Conclusions

The patients with acromegaly have a high prevalence of nodular thyroid disorders and thyroid dysfunction. No cases of thyroid cancer were found in our study. The periodic ultrasonography assessment of the thyroid is needed for evaluating patients with acromegaly.

## Introduction

Acromegaly is a chronic, debilitating endocrine disease caused by a growth hormone (GH)-secreting pituitary adenoma. The prevalence of acromegaly ranges between 2.8 and 14 per 100,000 people. It is usually associated with different thyroid disorders [1]. The exact prevalence of thyroid disorders among acromegalic patients varies across populations in different geographical areas [2,3]. Goiters are highly prevalent in the general population, with an age-related frequency as high as 74.2% in subjects aged 55-75 years and 54% in patients aged 76-84 years [3,4]. The prevalence of thyroid structural abnormalities in acromegaly ranged from 20% to 90% [5-7]. The underlying pathogenesis of goiter in acromegaly is not completely understood. Presumably, in patients with acromegaly, the pathogenesis of goiter is attributed to an increased level of insulin-like growth factor 1 (IGF-1), which binds to its specific receptor, expressed in thyrocytes [4].

Several studies have shown an inverse correlation between thyroid-stimulating hormone (TSH) levels and thyroid volume [5]. Moreover, studies have shown a direct relationship between the development of goiter and GH and IGF-1 levels or the duration of acromegaly [7-9,10]. The expression of the IGF-1 receptor in thyroid follicular cells implies that the interaction between TSH and IGF-1 might have a synergistic impact on the proliferation and growth of thyroid cells [11]. By contrast, thyroid cancer is rare. However, some studies show that the prevalence of thyroid cancer is slightly increased compared to the general population and can range from 4.7% to 5.6% of patients, represented mostly by the papillary variant [12-14]. The frequency of thyroid autoimmunity in acromegaly is comparable to that of the general population [6]. However, the occurrence of thyroid dysfunction is lower than that reported in the general population [15]. The relationship between acromegaly and various thyroid gland abnormalities has been recognized for a long time. However, the exact prevalence of thyroid abnormalities in individuals with acromegaly in our region has not been thoroughly investigated. Therefore, evaluating these changes and establishing monitoring and follow-up methods for these patients would facilitate the process for improved diagnosis, early identification, and treatment of disease-related abnormalities.

## Materials and methods

Objectives

In the present retrospective study, we aimed to provide a comprehensive description of the clinical, morphological, and biochemical features of thyroid disorder in Saudi patients with acromegaly and to establish its correlation with the activity and duration of the disease.

Patient selection

The study retrospectively examined and reviewed the medical records of 40 patients diagnosed with acromegaly who were receiving treatment at the outpatient endocrine clinic of the Obesity, Endocrine, and Metabolism Center (OMEC) at the King Fahad Medical City (KFMC) in Riyadh. The study included all patients with acromegaly, older than 14 years. The exclusion criteria included patients lost to follow-up and those with inadequate information in the clinical record. This study was approved by the research ethics committee of the KFMC Research Center (approval number: 23-025).​​​​​​

Data collection

The individuals included in the study had previously been diagnosed with acromegaly prior to their inclusion. The diagnosis of acromegaly was based on typical clinical features and laboratory data. Active disease was defined by the presence of elevated IGF-1 values for age and sex and unsuppressed GH levels below 1 ng/mL following a glucose suppression test (oral glucose tolerance test {OGTT}: 75 g oral glucose load) [16]. The cure was defined as a normal value for IGF-1 for age and sex and a mean GH value of less than 1 ng/mL as a result of previous surgery or radiation therapy [16]. Controlled disease was considered to have a normal IGF-1 value for age and sex and a mean GH value of less than 1 ng/mL obtained with medical therapy, irrespective of previous therapy [16]. The duration of disease activity was estimated from the reported date of onset of the disease to the date of the normalization of serum IGF-1 after treatment. The data collection involved extracting information from the electronic medical record system Epic (Epic Systems Corporation, Verona, WI) of the KFMC database. This included demographic data (age and gender), clinical evaluation, and details of hormonal levels (serum TSH, thyroxine {FT4}, triiodothyronine {FT3}, and IGF-1). Additionally, autoimmune markers (thyroid peroxidase antibodies {TPOAb} and thyroglobulin antibodies {TgAb}), thyroid morphology as determined by thyroid ultrasound (US), and the cytology of any thyroid nodules fulfilling the criteria for fine needle aspiration (FNA) were also included.

Statistical analysis

The data was analyzed using the Statistical Package for Social Sciences (SPSS) version 25 (IBM SPSS Statistics, Armonk, NY). Categorical variables were presented as a number (percentage), and quantitative data were presented as the mean (±standard deviation) or median (±standard deviation). The statistical significance of all statistical tests was determined by using a p-value of <0.05, while a 95% confidence interval was used for drawing statistical inferences. The intergroup comparison of the non-normal variables was done using the Mann-Whitney U test, whereas the normally distributed data was compared using Student’s t-test. A chi-square test was applied to measure the association between all the categorical variables and the underlying dependent variable.

## Results

The summary of the key features of the study population is presented in Table 1. Forty cases of acromegaly patients (27 males and 13 females) were included in this study, with a mean age of 46.78 ± 13.76 years. The estimated duration of the disease is 8.08 ± 6.43 years. The diagnosis of acromegaly was established by observing the distinct clinical manifestations of excessive growth hormone secretion, along with the elevated levels of serum IGF-1 and serum GH of greater than 1 ng/mL. In cases where serum IGF-1 levels were equivocal, an OGTT was conducted to further assess serum GH concentrations. Acromegaly was invariably attributed to GH-secreting adenoma. Hypophysectomy was performed on 37 patients (92.5% of the total), while radiotherapy was administered to 11 patients (27.5%). Thirty-one patients (77.5%) received medical therapy as monotherapy or combination therapy due to persistent disease following surgery, whereas three patients were on medical treatment as primary therapy. Twenty-seven patients (69.2%) had cured or controlled disease, while the remainder, 12 (30.8%), still had active disease.

**Table 1:** Clinical characteristics of the 40 acromegalic patients GH: growth hormone

Patient characteristics	
Age	46.78 ± 13.76 years
Sex	Male 27 (67.5%)
Acromegaly duration	8.08 ± 6.43 years
Acromegaly control	27 (69.2%)
Treatment	
Surgery	37 (92.5%)
Radiotherapy	11 (27.5%)
Medical therapy	31 (77.5%)
Sandostatin LAR alone	15 (48.4%)
Sandostatin LAR and GH antagonist	9 (29%)
Sandostatin LAR and cabergoline	7 (22.5%)

Goiter as estimated by ultrasound was found in 28 patients (70.0%); of these, 20 patients (71.4%) had multinodular goiter (MNG), four patients (14.2%) had solitary thyroid nodules, and four patients (14.2%) had thyroid cysts. Out of 28 patients with nodular goiter, FNA was performed in six patients as their ultrasound features fulfilled the criteria needed for FNA. Using the Bethesda classification system, five were benign follicular cells (Bethesda II), and one was a follicular lesion of unknown significance (Bethesda III); unfortunately, this patient declined to undertake further diagnostic evaluations. Referring to all patients (40), no cases of thyroid cancer were identified in our study. Among the 34 patients analyzed, 26.5% (9/34) had autoimmune thyroid disease for which autoimmune markers were available; the prevalence was 55.6% among females with a mean age of 52.8 years; in contrast, it was 44.4% among males with a mean age of 53.2 years. Abnormal thyroid function tests were found in 16 out of 40 patients (40.0%); in all 16 cases, primary hypothyroidism was the underlying cause. Positive thyroid autoimmunity was also present in four of the patients (25%). This study did not report any cases of central hypothyroidism or hyperthyroidism. The frequency of autoimmune thyroid disease among patients exhibiting abnormal thyroid morphology on thyroid ultrasounds was found to be similar to that of patients with normal thyroid US morphology: 14.3% (n = 4) versus 17.6% (n = 5). The frequency of thyroid alterations is detailed in Table 2.

**Table 2:** Frequency of thyroid alteration in 40 patients with acromegaly MNG: multinodular goiter

Thyroid alteration	Acromegalic patients
Normal	12/40 (30.0%)
Goiter	28/40 (70.0%)
MNG	20/28 (71.4%)
Single thyroid nodule	4/28 (14.2%)
Thyroid cyst	4/28 (14.2%)
Fine needle aspiration biopsy	
Benign follicular cells (Bethesda II)	5/6 (83.3%)
Follicular lesion of unknown significance (Bethesda III)	1/6 (16.6%)
Positive autoimmunity	9/34 (26.5%)
Hypothyroidism	16 (40.0%)
Hyperthyroidism	0 (00.0%)

According to disease activity, the analysis of thyroid morphology and volume was weakly correlated with age (r = 0.42; p = 0.01). No correlation was found between thyroid volume and sex, acromegaly duration, or IGF-1 level. Furthermore, data pertaining to the identification of primary thyroid disorders and their correlation with the diagnosis of acromegaly are inadequate. The patients with goiter and abnormal thyroid morphology (n = 28) were more likely to be males (64.3%; n = 18) and older (51.5 versus 45.9 years old) than females.

## Discussion

Acromegaly is often associated with structural changes in the thyroid gland, and it can be diffuse or multinodular [17]. The recent increase in the occurrence of abnormal thyroid structure may be attributed to the extensive use of highly sensitive thyroid ultrasonography. A hospital-based study conducted in Saudi Arabia found that 44% of patients had multinodular goiter (MNG) [18]. A further study showed that the frequency of thyroid nodules in the Saudi population exceeded 40% [19].

Our study confirms the high prevalence of goiter at 70%, including multinodular goiter at a rate of 71.4%, solitary thyroid nodules at a rate of 14.2%, and thyroid cysts at a rate of 14.2%. The findings are consistent with a previous study carried out in Brazil, which documented a prevalence rate of 71% [7], as well as a multicenter study undertaken in Italy, which found a prevalence rate of 78% [20]. The meta-analysis included a total of 22 studies, revealing a cumulative prevalence rate of 59.2% for thyroid nodular disease [21]. The prevalence of goiter in individuals with acromegaly indicates a considerable role of IGF-1 in the growth of the thyroid gland. This growth is a consequence of the continual stimulation of thyrocytes by GH and IGF-1 [10,22,23].

The role of TSH in the development of goiter is well recognized. This may be attributed to the synergistic impact of IGF-1 and TSH on thyrocytes [24]. Although acromegalic patients frequently develop goiter, thyroid cancer continues to be a contentious issue [13,23]. In this study, no cases of thyroid carcinoma were reported. Nevertheless, research has shown that thyroid cancer affects approximately 3%-7% of patients, with the papillary variant constituting the majority [8,13,25,26]. The scientific literature [27,28] suggests that this may be the result of IGF-1 promoting neoplastic angiogenesis, metastasis, and cancer cell proliferation. The aforementioned may be due to the methodological factors of the study, such as a limited number of cases, and the true prevalence of thyroid cancer among our patients may be underestimated.

Importantly, our study showed that structural thyroid abnormalities were observed more in older patients (r = 0.42; p = 0.01). This finding is consistent with the same finding reported in another study, which showed that thyroid multi-nodularity was more commonly seen in older patients [29]. Despite the high prevalence of goiter in acromegaly, there is no significant correlation between the activity of acromegaly, as indicated by the IGF-1 value, and any alterations in the volume or morphology of the thyroid gland in our patients. This finding is consistent with previous studies [6,7]. Nevertheless, a study conducted on a large population has shown a correlation between the concentration of IGF-1 and the volume of the thyroid gland [30]. Furthermore, other studies have shown a reduction in thyroid volume after the normalization of IGF-1 levels [8,22]. This finding highlights the role of IGF-1 as a crucial element in the development of goiter in people with acromegaly. In contrast to the findings reported by other studies [10,31], our study did not find any association between goiter development and disease duration.

The majority of individuals with acromegaly have normal thyroid functioning, while a subset may experience hypothyroidism. A greater proportion of these individuals develop secondary hypothyroidism, which may be attributed to the occurrence of the tumor or as a result of surgical intervention and radiation therapy. Primary hypothyroidism has been seen in acromegaly patients, perhaps as a consequence of the high incidence of thyroid autoimmunity [15]. The effect of IGF-1 and GH on thyroid function leads to a combination of several abnormalities in thyroid function as well. Primary hypothyroidism was the most prevalent thyroid condition seen in our study, affecting 40.0% of the patients. The prevalence reported is considerably higher than those published by other authors, which range from 25% to 33.6% [15,16]. In comparison to the general population, a study conducted in Saudi Arabia revealed a prevalence rate of 29.1% for hypothyroidism among a sample size of 3,872 individuals [32]. A different study also carried out in Saudi Arabia included 454 individuals aged 21-60 years old. Hypothyroidism was found in 25.5% of the responders [33].

In our study, we did not find any cases of secondary hypothyroidism. However, a higher proportion of secondary hypothyroidism was documented in another study, perhaps attributed to advancements in surgical techniques, the availability of medical treatments, and a lower rate of reoperations and radiation therapy in our cohort [15]. The prevalence of thyroid autoimmunity was found to be 26.5% in the whole group, with 55.6% in females and 52.8% in males. While there is a paucity of reference data specifically for the Saudi population, it seems that the prevalence of thyroid autoimmunity is significant among our acromegalic patients. This observation has also been confirmed in another study, where 17.1% of the participants had positive thyroid autoimmunity [15]. A thorough study, which included 258 individuals with acromegaly, revealed a 23% prevalence of thyroid peroxidase antibodies (TPOAb) and a 21% prevalence of thyroglobulin antibodies (TgAb) [7]. Nevertheless, some studies have reported a lower frequency rate. For instance, in an Italian study including 28 acromegalic patients, Cannavò et al. showed that only 7% of them tested positive for TPOAb [6]. Similarly, Rogozinski et al. reported a low prevalence of 9% for TPOAb in their study of 34 Argentinean acromegalic individuals [34].

The primary constraint of our study is the limited sample size, which includes patients spanning a broad range of ages. Consequently, the findings may not accurately represent the actual prevalence of thyroid disorders in individuals with acromegaly. In addition, the thyroid ultrasound was performed and analyzed by different physicians. Moreover, the study has a retrospective design, and not all patients were subjected systematically to a fine needle aspiration biopsy. Additionally, there was no control group included in the study.

## Conclusions

This study has confirmed the higher frequency of structural thyroid diseases among individuals with acromegaly. The prevalence of thyroid autoimmunity has increased, perhaps playing a role in the development of thyroid disorders in individuals with acromegaly. The thyroid morphology, as evaluated by ultrasound, is not correlated to circulating levels of GH and/or IGF-I or the duration of the disease. The finding provides compelling evidence that emphasizes the need for regular clinical, biochemical, and ultrasonographic evaluations for patients diagnosed with acromegaly with regard to their thyroid glands.
